# Volatile Organic Compound Metabolism on Early Earth

**DOI:** 10.1007/s00239-024-10184-x

**Published:** 2024-07-17

**Authors:** S. Marshall Ledford, Laura K. Meredith

**Affiliations:** 1 Genetics Graduate Interdisciplinary Program, University of Arizona, Tucson, AZ 85721 USA; 2 School of Natural Resources and the Environment, University of Arizona, Tucson, AZ 85721 USA; 3 BIO5 Institute, University of Arizona, Tucson, AZ 85721 USA

**Keywords:** LUCA, VOC, Atmosphere, Trace gas, Metabolism, Volatile metabolite, Biosignature

## Abstract

Biogenic volatile organic compounds (VOCs) constitute a significant portion of gas-phase metabolites in modern ecosystems and have unique roles in moderating atmospheric oxidative capacity, solar radiation balance, and aerosol formation. It has been theorized that VOCs may account for observed geological and evolutionary phenomena during the Archaean, but the direct contribution of biology to early non-methane VOC cycling remains unexplored. Here, we provide an assessment of all potential VOCs metabolized by the last universal common ancestor (LUCA). We identify enzyme functions linked to LUCA orthologous protein groups across eight literature sources and estimate the volatility of all associated substrates to identify ancient volatile metabolites. We hone in on volatile metabolites with confirmed modern emissions that exist in conserved metabolic pathways and produce a curated list of the most likely LUCA VOCs. We introduce volatile organic metabolites associated with early life and discuss their potential influence on early carbon cycling and atmospheric chemistry.

**Supplementary Information:**

The online version contains supplementary material available at 10.1007/s00239-024-10184-x.

## Introduction

From its origin, life has influenced the environment through various metabolic interactions. Physically this is shown in the geological record, with the earliest evidence indicating biologically derived ^13^C depletion in sediments dating 3.6–3.8 Gya (Arndt and Nisbet 2012; Rosing 1999). Paleontological evidence shows the potential for aquatic microbial mats at 3.4 Gya and some evidence indicates terrestrial biofilms at ~ 3.0 Gya (Tice and Lowe 2004; Thomazo et al. 2018; Homann et al. 2018). Numerous studies have sought to understand these early metabolic interactions through the predicted metabolism of the last universal common ancestor (LUCA). LUCA is defined as the oldest traceable period of vertical gene transfer and marks the onset of Darwinian evolution (Doolittle and Brown 1994; Weiss et al. 2018). While often limited by the large scale of evolutionary time that has passed between LUCA and the extant genomes or proteomes used to infer it, diverse methods have been used for examining genes and protein functions that existed in LUCA. To this end, highly conserved metabolic pathways have been broadly attributed to LUCA, including amino acid, carbon, and energy/cofactor metabolism (Crapitto et al. 2022; Goldman and Kacar 2021). The extent and form of LUCA metabolites’ influence on early biochemistry depends on a variety of factors, notably their physical or chemical properties and how those relate to environmental context. Unique among these characteristics is volatility, defined by the tendency of a compound to partition into the gas vs condensed phase (Finlayson-Pitts and James 1987). Volatile organic compounds (VOCs) are noted for their diverse structures and functions but are generally low molecular weight (LMW; ~ 50–200 Da) and have appreciable vapor pressures under ambient conditions (Rowan 2011). VOCs exhibit unique inter- and intra-ecosystem interactions. In terrestrial systems, they act as sources of information and energy exchange within the porous soil matrix and have major roles in carbon cycling (Honeker et al. 2021). While most data on terrestrial VOCs emphasize plant-driven interactions, some soil communities may be more analogous to that of early terrestrial life (Thomazo et al. 2018; Thomazo et al. 2020). Biology is also believed to be a main driver of VOC production and consumption in ocean systems (Dixon et al. 2014; Royer et al. 2016; Halsey et al. 2017; Halsey and Giovannoni 2023). The largest oceanic sink for some VOCs is surface metabolism influenced by ambient atmospheric VOC concentrations and dissolved organic matter (DOM) availability (Phillips et al. 2021). While the oceans are considered a net source of many atmospheric VOCs, the processes by which VOCs are emitted from marine environments are complex and depend on a variety of abiotic and biotic factors that are not well constrained (Yu and Li 2021; Pozzer et al. 2022). Therefore, flux rates may vary drastically. Marine microorganisms may produce different sea–atmosphere flux rates depending on the availability of terrestrial inputs by proximity, for example, at coastal vs open ocean sites (Sauer 1981). The difficulty in isolating aquatic sources from terrestrial inputs makes understanding strict oceanic contributions to global VOC fluxes difficult. Still, metabolism by aquatic species, notably phytoplankton, is an important driver of VOC flux at the sea–atmosphere interface—one route of exchange connecting aquatic and terrestrial biology (Fink 2007; De la Porte et al. 2020). As reactive atmospheric trace gases, biogenic VOCs influence the lifetime of greenhouse gases, ozone pollution formation, and organic aerosol production (Chameides et al. 1988; Laothawornkitkul et al. 2009; Park et al. 2013; Müller et al. 2017) and therein VOCs exert an important biological influence on the environment.

VOC metabolism may have developed early in life’s history. It is believed that early heterotrophs depended on the availability of low-molecular weight metabolites for the extraction of organic matter from the environment prior to the evolution of exoenzymes (2.8–2.5 Ga; Mahmoudi et al. 2023; Gruen et al. 2019). Given the inverse relationship between molecular weight and vapor pressure, it is possible that some of these low-molecular weight compounds had appreciable volatility. Many modern volatiles are readily produced through the photochemical degradation of larger biomolecules (Moran and Zepp 1997), among other processes (Hädeler et al. 2023), making even nonvolatile biogenic metabolites potential sources of VOC production through abiotic means. Beyond this, LUCA metabolic pathways, including energy, nitrogen, and carbon metabolism, contain inorganic volatile metabolites (e.g., CO_2_, N_2_, CO). In modern species, many of these pathways also include VOCs as intermediates (Schmidt et al. 2015). Given this evidence and that we have yet to identify a species which does not metabolize VOCs (Meredith et al. 2023), it is reasonable to consider that VOC metabolism developed early in life’s history.

VOC cycling on early Earth may have been different from what is observed today. The primary destructive mechanism of modern VOCs is the oxidizing atmosphere in which VOCs are rapidly degraded (Lloyd et al. 1976; Shu and Atkinson 1995; Atkinson and Arey 2003). The atmospheric chemistry may have been different during the Archaean eon, prior to the great oxidation event (GOE), and in an atmosphere described as slightly reducing (Catling and Zahnle 2020; Holland 2006). Under these conditions, VOC sources to the atmosphere may have also been different as it has been demonstrated that anoxic conditions can result in significant increases in some VOC emissions from modern soils, driven by a shift toward VOC containing anaerobic metabolic pathways or a reduction in atmospheric oxidation, or both (Bourtsoukidis et al. 2018; Jiao et al. 2023; Pugliese et al. 2023). Modeling acetaldehyde, methanol, and isoprene in anoxic exoplanet atmospheres has shown the potential for VOCs to build to concentrations beyond what we experience on Earth today (Huang et al. 2022; Zhan et al. 2021), sometimes driving the development of secondary features, such as carbon monoxide runaway or an organic haze (Zhan et al. 2022; Arney et al. 2018). Modern biogenic VOCs are well characterized for contributing to the development of organic aerosols, which influence cloud formation and solar radiative balance and are a key factor in the production of blue haze present on some heavily forested mountains (Claeys et al. 2004). Intermittent organic haze formation throughout the Archaean eon may have been possible (Zerkle et al. 2012; Izon et al. 2007). Formation of an organic haze layer has been investigated as a mechanism for protecting the earliest life from UV radiation prior to ozone (Sagan 1973) and shielding greenhouse gasses (notably ammonia) from photodissociation, highly dependent on atmospheric composition (Trainer 2013; Sagan and Chyba 1997; Pavlov et al. 2001). Atmospheric VOCs produced through methane photochemistry could have contributed to this organic haze and been a source of organic carbon in early oceans (Kasting et al. 1989; Cockell and Knowland 1999; Haqq-Misra et al. 2023). Therefore, VOCs produced abiotically could have been directly accessible for metabolism. VOCs tend to increase the lifetime of methane in the atmosphere by competing for reaction with hydroxyl radicals (⋅OH) and likewise enhance the radiative forcing of methane, inducing a positive bVOC-OH-CH_4_ feedback loop (Boy et al. 2022). Therefore, VOCs may have had an influence on Archaean atmospheric chemistry and climate dynamics through impacts on atmospheric oxidative capacity, haze formation, and methane chemistry. Still, even with the knowledge that metabolic activity is the main driver of VOC cycling today, the direct contribution of biology to early Earth non-methane gas-phase organics remains unexplored.

Here, we aim to identify the VOCs that may have been cycled by early life. By assessing predicted LUCA enzyme functions, we confirm the metabolism of numerous VOCs in LUCA and constrain precisely the compounds that may have acted as biologically relevant trace gases in the Archaean atmosphere. We describe this early volatile metabolome (“volatilome”) and how it might have served as an important interface for biosphere-mediated carbon cycling (Meredith and Tfaily 2022). By proposing VOCs as metabolically relevant trace gas atmospheric constituents during the Archaean, we provide an additional perspective on potential biological mechanisms influencing global atmospheric chemistry in Earth’s pre-oxygenated atmosphere and inform on an important aspect of carbon cycling on early Earth.

## Methods

### Identifying Metabolites Associated with LUCA

Analyses on LUCA rely on diverse methods that target particular proteomic and genomic features, such as protein domains, gene families, molecular functions, or protein structures (Crapitto et al. 2022). To identify VOCs associated with LUCA, we relied on the results from Crapitto et al. (2022), which collated LUCA predictions from eight literature sources spanning two decades (Harris et al. 2003; Mirkin et al. 2003; Delaye et al. 2005; Yang et al. 2005; Ranea et al. 2006; Wang et al. 2007; Srinivasan and Morowitz 2009; Weiss et al. 2016). In total, Crapitto et al. compiled 2230 COG (clusters of orthologous groups of proteins; Galperin et al. 2015) database identifiers as associated with LUCA and provided their corresponding literature source. While maintaining the connection to their primary literature source, we converted these LUCA COGs to enzyme commission (EC) numbers and linked these enzymes to metabolites through the Kyoto Encyclopedia of Genes and Genomes (KEGG; Kanehisa and Goto 2000) to then estimate the volatility of these LUCA protein linked metabolites (methods next section).

**Fig. 1:**
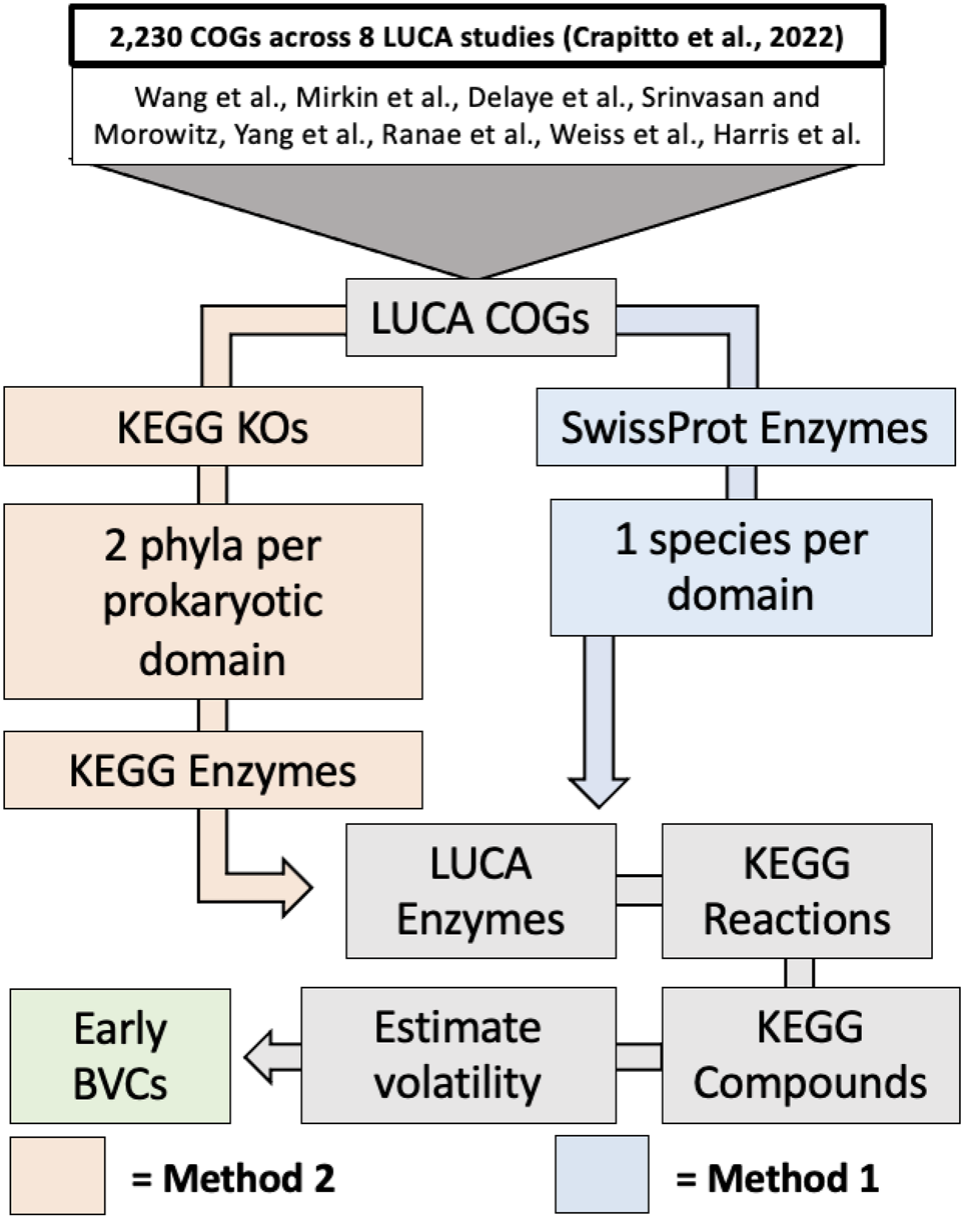
Overview for the broad approaches to determine volatile compound metabolism associated with LUCA. Method 1 and Method 2 refer to the COG–EC translation approaches

To convert from COG to EC number, we used two methods. In Method 1, we directly followed the methodology Crapitto et al. (2022) used to identify EC numbers for a subset of 366 COGs. We expanded the approach to all 2230 of their LUCA-associated COGs and manually searched the curated Swiss-Prot database to obtain EC numbers for all LUCA COG-related enzymes. EC numbers were only included if found in proteins spanning all three domains (Eukaryota, Archaea, and Bacteria). In Method 2, we followed a similar approach but used the KEGG database rather than Swissprot. Through the R package KEGGREST (Tenenbaum 2021), we searched each KEGG ortholog (KO) for a linked COG as listed in KEGG as an analogous outside database identifier. We filtered this list to select all LUCA COG-associated KOs. In a manner similar to Method 1 we evaluated the distribution of KOs across domains. For Method 2, we only included KOs present in at least two unique phyla in both bacteria and archaea, adapting the phylogenetic distribution criteria from Weiss et al. (2016). We considered KOs both associated with a LUCA COG and that met this distribution criteria to be appropriately translated from LUCA COG to LUCA KO. We then linked KOs to all of their enzymes using KEGGREST to, in an automated manner, output the EC number(s) listed for each KO (Tenenbaum 2021). Methods 1 and 2 each produced a list of potential LUCA-associated EC numbers translated from the original COG dataset (Fig. 1). They relied on two separate databases for this translation and have similar but unique phylogenetic distribution requirements. For both methods, we then exploited compound–enzyme associations through KEGG to identify the metabolites that are associated with LUCA enzymes. To do this, we generated a complete list of KEGG enzymes and all associated reactions and metabolites following methods described in Meredith et al. (2023). A metabolite was considered associated with an enzyme if it was a substrate in a reaction that enzyme catalyzed. Merging these data sets, we generated a final list of all potential LUCA COGs, enzymes, reactions, and metabolites for each of the eight LUCA studies.

### Predicting Metabolite Volatility

The newly developed R package, *volcalc*, uses the SIMPOL.1 method to estimate vapor pressure of KEGG compounds (Riemer and Scott 2023; Meredith et al. 2023; Pankow and Asher 2008). This is done following the equation:

$$\log_{10} P = b_{0} + \sum\limits_{k} {v_{k} b_{k} }$$

where *P* represents estimated vapor pressure, *b*_*0*_ is a constant, *b*_*k*_ is the functional group contribution term for group *k*, and *v* is the number of groups of type *k* in the compound. The vapor pressure is reduced with increasing carbon number. Vapor pressure is further modified in accordance with functional group additions decreasing *P* to varying degrees. Vapor pressure estimates are used to predict metabolite volatility by comparing the relative tendency to partition into the gas vs. condensed phase using the ideal gas law to calculate the mass-based saturation vapor concentration (µg m^−3^) (log_10_C* = log_10_(PM/RT)), where *M* is the molecular mass, *R* is the universal gas constant, and *T* is the temperature (Donahue et al. 2006). Thresholds for nonvolatile, intermediate volatility, and volatile are on the order *C*^∗^  = 0.01 μg m^–3^, 1 μg m^–3^, and 100 μg m^–3^, respectively, or on a log scale: log10*C*^∗^  =  ﻿−﻿ 2, 0, and 2. The result is a relative volatility index (RVI) value calculated for each compound. We adopted this RVI scale to then estimate volatility of all LUCA-associated compounds in the standard environment assumed in *volcalc* (v.1.02) in a non-polluted atmosphere. This considers compound volatilities according to the following RVI categorical values: nonvolatile (< − 2), low volatility (− 2 to 0), intermediate volatility (0–2), and high volatility (> 2). Compounds with a calculated volatility of “high” were considered to be volatile compounds.

### Compound Classification

To develop comparisons by compound type, we hierarchically classified all KEGG volatiles using the program ClassyFire through the R package *classyfireR* (Djoumbou Feunang et al. 2016). We downloaded KEGG metabolite IDs as csv files and then translated them through the online platform, The Chemical Translation Service, to International Chemical Identifiers (InChI Keys) as required by classyfireR (Wohlgemuth et al. 2010). We then used *classyfireR* to classify all KEGG compounds categorized as highly volatile through *volcalc* (Table S1). We merged these with the LUCA metabolite dataset to create a list of all KEGG volatiles and their classifications for LUCA-specific metabolites. To give context in modern species, we then compared these results to volatiles linked to all ~ 9000 organisms in KEGG, as well as to the average classification distribution found in each domain of life. We generated a list of all KEGG genomes and grouped them by domain (Tenenbaum 2021). We retrieved complete KO content per species and linked KOs to compounds through their associated enzymes to generate a complete volatile metabolite profile for each genome. We classified all of these volatiles and report the percentage of each species’ volatile profile belonging to each volatile subclass. For each of the 8 LUCA studies used for this analysis (Weiss et al. 2016; Harris et al. 2003; Mirkin et al. 2003; Delaye et al. 2005; Yang et al. 2005; Ranea et al. 2006; Wang et al. 2007; Srinivasan and Morowitz 2009), all predicted volatile compounds were grouped by subclass and similarly plotted to identify differences in volatile metabolome diversity and composition between modern and predicted ancient life.

### Confirmation of Volatility and Pathway Enrichment Analysis

While the previous analyses first provided a broad understanding of all potential LUCA volatiles, we sought to identify the specific VOCs that were the strongest candidates as LUCA metabolites and remove any potential false positives. First, we addressed the limitations of our vapor pressure estimation tool. It is possible that some predicted compounds, while volatile, may not translate to detectable emissions and interactions with the environment. *Volclalc* does not fully take into consideration some intermolecular forces (e.g., hydrogen bonding, dipole moments), and it has been documented that functional group addition vapor pressure estimation methods (like SIMPOL.1) tend to overestimate the volatility of compounds especially subject to these forces (e.g., leucine; O'Meara et al. 2014). For these reasons, all LUCA-associated compounds determined to have a high volatility were manually evaluated by searching the literature and the online microbial VOC (mVOC) database for detected emissions from modern species (Table S2; Lemfack et al. 2018**)**.

It is not always clear whether association of a COG to a given enzymatic class may ensure functional specificity to all plausible substrates linked with that enzymatic class. In this analysis, we were limited by our use of the KEGG database, and therefore enzymatic classes, to create linkages between COGs and VOCs. It may be the case that modern enzymatic classes (e.g., EC: 1.1.1.1; alcohol dehydrogenase) are associated with multiple genes that, while functionally related, do not share an evolutionary history and may associate with different substrates within the same class. In this case of convergent evolution, a particular enzymatic class may be overrepresented among taxonomic clades within KEGG. This may lead an enzymatic class to be linked with many volatile metabolites, only some of which are truly LUCA associated. In the above example, EC: 1.1.1.1 is linked with multiple reactions involving many different volatile alcohols. For the reasons described above, including all of these alcohols as “LUCA VOCs” may introduce false positives into our analysis. Here, we accounted for this by relying on the KEGG pathway mapping tool that links multiple substrates and enzymes that are known to be connected to each other along metabolic pathways. We reasoned that it would be unlikely for a LUCA enzyme to exist in isolation, without being a part of a broader metabolic function. For all of our LUCA-associated enzymes, we identified all KEGG pathways in which they exist. We then determined which KEGG pathways were enriched for LUCA enzymes. We determined enrichment by performing a hypergeometric test, which estimates whether a given KEGG pathway contains significantly more LUCA-associated enzymes than would be expected if were to be randomly drawn from a list of all metabolic enzymes in the KEGG database. We then manually searched each LUCA VOC to determine whether they were linked with a LUCA enzyme in one of these enriched pathways.

To be considered a LUCA-associated VOC through this more stringent requirement, a VOC would have to be linked with a LUCA enzyme and be a metabolic intermediate in association with that enzyme in a KEGG metabolic pathway. Finally, that metabolic pathway would need to be enriched in other LUCA enzymes as well. An example of this requirement is shown in Fig. S1. Here, VOCs associated with EC 1.1.1.1 meet the enrichment requirement if they are associated with EC 1.1.1.1 within the glycolysis pathway. This pathway contains many additional LUCA enzymes. VOCs associated with EC 1.1.1.1 in the chloroalkane and chloroalkene degradation pathway did not meet this requirement, as there were no other additional LUCA enzymes in this pathway.

## Results and Discussion

### Early Life Boasts a Diverse Potential Volatile Metabolome

**Fig. 2:**
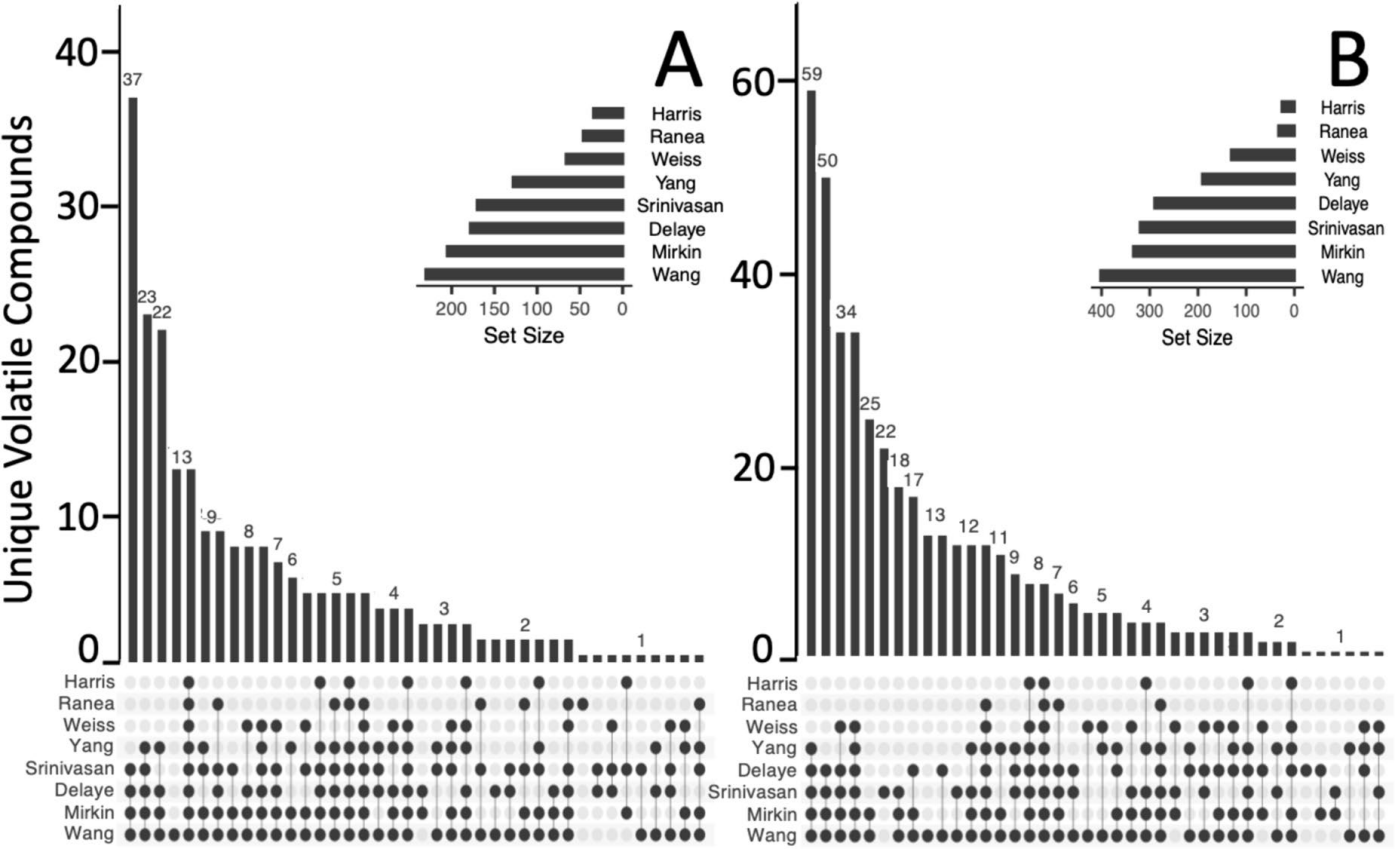
Distribution of predicted highly volatile LUCA enzyme-linked volatile metabolites across sources by two COGs to enzyme conversion methods: **A** Method 1 adaptation of Crapitto et al. (2022) (*n* = 237). **B** Method 2 adaptation to Weiss et al. (2016) (*n* = 477)

We identified numerous putative volatile metabolites associated with LUCA. Across all 2230 LUCA-associated COGs summarized by Crapitto et al. (2022). Method 1 (manual Swissprot based enzyme curation) identified 381 complete ancestral enzymes linked to 817 unique KEGG compounds, of which 237 had vapor pressures estimated as “highly volatile.” Wang et al. (2007), Mirkin et al. (2003), Delaye et al. (2005), Srinvasan and Morowitz (2009), and Yang et al. (2005) were each linked to a COG associated with at least 50% of the volatiles in this dataset, while Ranea et al. (2006), Weiss et al. (2016), and Harris et al. (2003) each had less, highlighting much cross-study variability (Fig. 2A). Specificity of VOCs to particular datasets is likely driven by their diverse study methods. For example, of the 2,230 total COGs identified by Crapitto et al., only 100 were attributed to Harris et al. who focused largely on universal genes related to the transfer of genetic information. Our COG to enzyme translations agreed with this, as the KEGG Aminoacyl-tRNA biosynthesis pathway contained more of the 73 Harris enzymes than any other KEGG pathway. Aminoacyl-tRNA synthetases were one of the gene groups specifically discussed in Harris et al. (2003). In contrast, the dataset from Wang et al. contained over 1300 COG IDs and consequently, a much larger list of related enzymes and VOCs. Differences in these datasets have been thoroughly explored in Crapitto et al. (2022).

Method 2 (KEGG-based COG/KO to EC number translation) produced even more volatiles, with 848 unique enzymes associated with 1442 KEGG compounds, of which 477 were categorized as highly volatile (Fig. 2B). Most volatile compounds were found in more than one study (92% and 94% for Methods 1 and 2, respectively), and few volatiles were only associated with one study (14 and 35 in Methods 1 and 2, respectively). For both methods’ volatile predictions, the largest subclass of volatile compounds was “amino acids, peptides, and analogues.” Beyond this, Method 2 identified 25 carbonyl compounds (vs Method 1, only 8) and 11 fatty acids/conjugates (vs Method 1, only 3) as its next largest volatile subclasses. The second and third largest volatile subclass groups via Method 1 were carbohydrates/carbohydrate conjugates and short-chain keto acids/derivatives. There were 0 terpenoids identified via Method 1, while Method 2 identified a total of 7 (6 monoterpenoids and 1 sesquiterpenoid). Lists of all predicted LUCA volatile compounds, their linkage method, and their associated sources are available in Table S3.

**Fig. 3:**
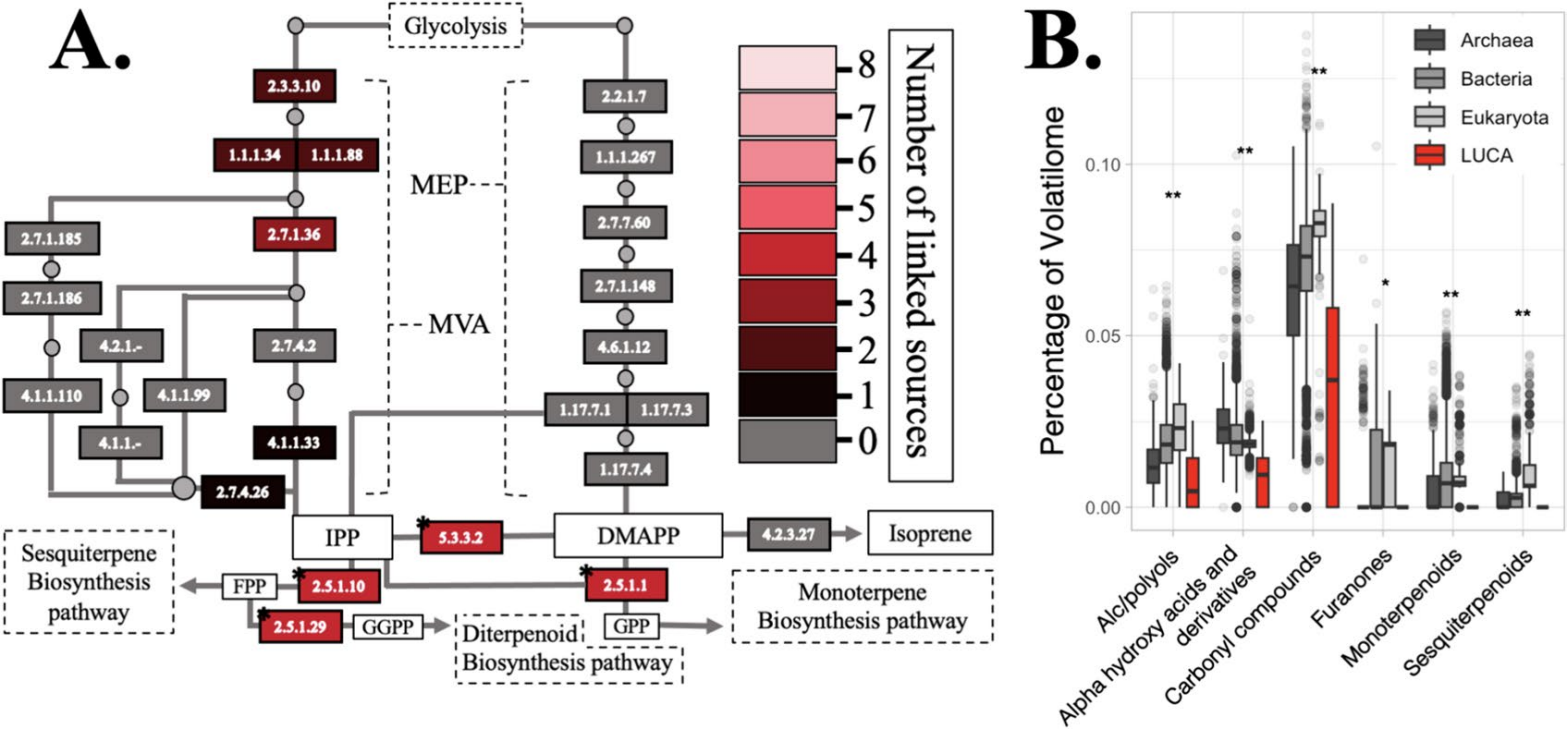
Broad assessment of potential LUCA volatiles. **A** Distribution of LUCA enzymes across the KEGG terpenoid backbone biosynthesis pathway regardless of method. Circles represent metabolic intermediates. Notable intermediates are highlighted through solid white rectangles. Enzymes are color coded by the number of papers with a linked COG. Dashed rectangles represent pathways. Asterisks indicate enzymes also in the common enzyme list. **B** Frequency of KEGG volatile compound classes across modern domains and LUCA papers from the common list (archaea, *n* = 411; bacteria *n* = 7860; eukaryota *n* = 926; LUCA *n* = 8). Asterisks indicate significant difference (*p* value < 0.05, wilcoxon test) when comparing LUCA vs the average of modern domains

Volatile terpenoids are the most diverse and highly emitted (by mass) class of VOCs today, and we found limited evidence for terpenoid metabolism in LUCA. Terpenoid biosynthesis occurs in three major stages (Hoshino and Villanueva 2023) as illustrated in the terpenoid backbone biosynthesis pathway (map00900, Fig. 3A). The first stage involves the production of two precursors: isopentenyl diphosphate (IPP) and its isomer dimethylallyl diphosphate (DMAPP; Carretero-Paulet et al. 2002). These can be produced through either the methylerythritol phosphate (MEP) or mevalonate (MVA) pathways (Kuzuyama and Seto 2012). Accounting for any enzyme found through either method permits a nearly complete MVA pathway route, missing only a single enzyme out of six, albeit only within particular LUCA studies. The enzyme responsible for the conversion between IPP and DMAPP was predicted to be in LUCA and is associated with COGs spanning 4 LUCA studies. The second stage of terpenoid biosynthesis involves three direct terpenoid precursors: GPP, FPP, and GGPP (Hoshino and Villanueva 2023). Each of these precursors was also linked with a LUCA COG. Therefore, the very broad approach includes 10/11 enzymes necessary to produce all major terpenoid secondary metabolite pathway precursors through the MVA pathway. Interestingly, previous work has highlighted the MVA pathway as likely ancestral, over MEP (Lombard and Moreira 2011); however, this remains an active area of debate (Hoshino and Gaucher 2018). Enzymes in monoterpenoid, diterpenoid, triterpenoid, and sesquiterpenoid biosynthesis pathways were absent. Although Method 2 identified 6 volatile monoterpenoids and 1 volatile sesquiterpenoid, these compounds were often linked with a generalist enzyme (e.g., 1.2.1.3, aldehyde dehydrogenase) and lacked contextual support, i.e., the pathways the enzymes were found in (e.g., map00981, insect hormone biosynthesis) did not include additional LUCA enzymes.

We found that the two methods shared a set of 276 LUCA-associated enzymes linked to 206 potential volatile metabolites, here described as the common list. This more narrow approach garners greater certainty in results by relying on more than one method for associating LUCA COGs with enzymes and reducing false positives which may persist in either Method 1 or 2. Through the common enzyme list, we no longer observed key enzymes involved in terpenoid production, including no enzymes of the MVA or MEP pathways. There was also a complete lack of terpenoid secondary metabolite biosynthesis enzymes, and we did not identify any LUCA-associated volatile monoterpenoids, diterpenoids, triterpenoids, or sesquiterpenoids. We observed enzymes needed for conversion between IPP and DMAPP and production of all precursors needed for secondary terpenoid biosynthesis pathways. Yet overall, we found a significant depletion of the terpenoid backbone biosynthesis pathway, in agreement with other work (Crapitto et al. 2022). Using this common list, we compared distributions in volatile metabolite classification profiles of LUCA against modern species across domains (archaea, bacteria, and eukaryota) (Fig. 3B). Alcohols, furanones, terpenoids, and carbonyl compounds represented a smaller percentage of LUCAs volatilome when compared with modern species. In contrast, amino acids, peptides, and analogues made up a higher percentage. This highlights that ancient VOC metabolism may have been dominated by highly conserved metabolites involved in primary metabolism, with limited terpenoids and other secondary metabolites (Table S4).

### A Conservative List of LUCA VOCs

**Fig. 4:**
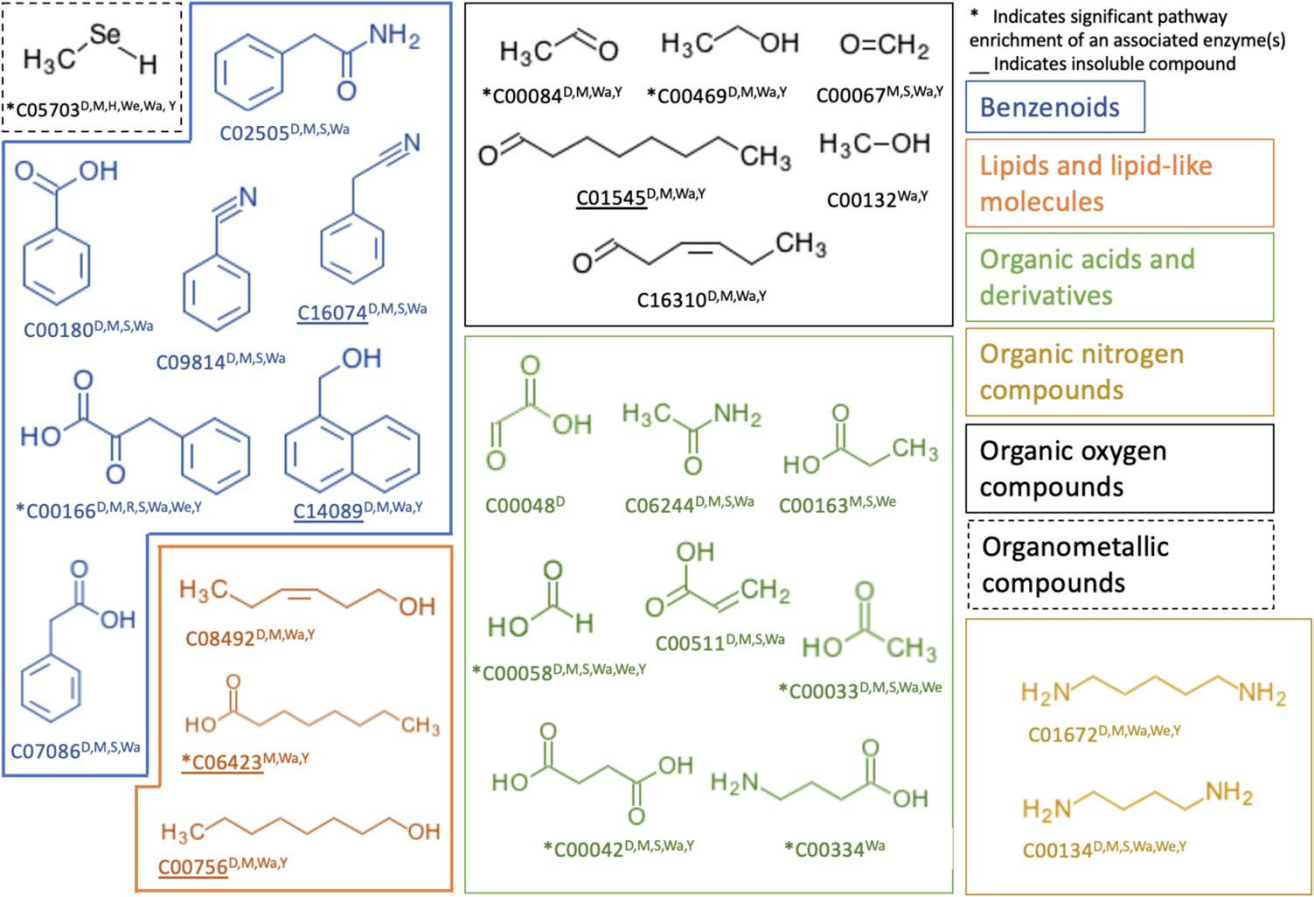
Early life-associated compounds with confirmed emissions from biological sources. KEGG Compound ID and ClassyFireR superclass are indicated. Compound associations with each LUCA study are indicated in an abbreviated superscript: D (Delaye), M (Mirkin), H (Harris), R (Ranea), S (Srinivasan), We (Weiss), Wa (Wang), and Y (Yang). Asterisks indicate a direct substrate of an enzyme in a pathway significantly enriched for common LUCA enzymes (hypergeometric test). Underlined compounds are determined to be very slightly soluble or insoluble in water at STP

The previous analyses broadly address volatile compounds that may be linked to an enzyme that we associate with a LUCA COG. This starting point allows us to observe overall trends in ancient volatile metabolism. We next created a conservative list of compounds that we have the highest confidence in as VOCs cycled by LUCA, based on two major criteria: (1) confirmed emissions by modern species and (2) existence in a broader metabolic context within LUCA. Of 206 compounds from the common list of putative LUCA volatiles, 20 (10%) were not organic and included water, ammonia, and CO_2_. Of the remaining 186, 27 (15%) have detected emissions from modern biogenic sources and represent our conservative list of LUCA VOCs (Fig. 4). For this subset, we identified six (formic acid, acetaldehyde, acetic acid, formaldehyde, ethanol, and methanol) that have available net global biogenic flux data (Sindelarova et al. 2014; Guenther et al. 1995). We highlight that for these six compounds associated with ancient life, there are appreciable modern day biogenic global emissions into the atmosphere of greater than ~ 179 (Tg/year^−1^), a similar order of magnitude to modern methane production from all sources (~ 500–600 Tg/year^−1^; Dlugokencky et al. 2011). Due to knowledge limitations of ancient biomass, it is difficult to estimate what this flux may have looked like on early Earth. Still, early life’s association with these highly volatile compounds with large modern net fluxes at the regional and global scale indicates that these ancient VOCs may have been influential on carbon cycling and had a significant impact on the Archaean environment.

The pathways significantly enriched for LUCA enzymes and associated with LUCA VOCs are consistent with current understanding of LUCA metabolism. For example, glycolysis, gluconeogenesis, carbon fixation, the citrate cycle, and various amino acid biosynthesis and metabolism pathways have established roots in LUCA or even earlier (Braakman and Smith 2012; Harrison and Lane 2018; Sumi and Harada 2021) and in them, we find many LUCA enzymes with VOCs as direct substrates. A complete list of pathways enriched for LUCA enzymes is provided in Table S5. For the 27 LUCA VOCs with known emissions in modern species, 9 were associated with enzymes as intermediates in pathways significantly enriched for LUCA enzymes (hypergeometric test; R Core Team 2021). Of these nine VOCs, two (4-Aminobutanoic acid and octanoic acid) were linked with COGs predicted to be LUCA associated in fewer than half of the eight cited LUCA studies. We consider the remaining seven VOCs: acetic acid, ethanol, formic acid, acetaldehyde, methaneselenol, succinic acid, and phenylpyruvate, to have the strongest evidence as LUCA associated (Table 1). These compounds were associated with COGs across at least four LUCA studies, were linked to enzymes via two different COG to enzyme translation approaches with taxonomic distribution requirements, are confirmed to be emitted by modern species, and serve as intermediates in KEGG pathways that are significantly enriched across LUCA enzymes. Several of these have been previously noted as potential early metabolites (Hernández-Montes et al. 2008; Fuchs 2011; Xavier et al. 2021; De Graaf et al. 2023), albeit not from the perspective of their impact as VOCs. It is certainly possible, and even likely, that many other VOCs were also metabolized by LUCA, but these are the seven for which we have the most confidence. While we cannot make more definitive claims about the other metabolites listed in this study, we still see that their metabolism is highly conserved across modern domains. Therefore, many of these compounds may be associated with *early life*, even if not definitively within LUCA. This may be especially the case for dominant modern volatiles, such as methanol or acetone, which did appear in the data, but did not meet our criteria of existing in half of the LUCA studies or were only identified via one translation method. Another example, putrescine (C00134), was linked with six LUCA papers across both COG-enzyme conversion methods and is associated with a core metabolic function as an intermediate in the arginine and proline metabolism pathway (map00330). However, this pathway was not enriched for LUCA enzymes, excluding even this likely ancient metabolite from our final strict dataset. This highlights the conservative nature of our filtering and the need to still consider these additional metabolites for future analysis.

**Table 1:** Modern bVOCs found in enriched LUCA pathways

KEGG pathway	*p* value	bVOCs	VOC enzymes	LUCA enzyme coverage
map00620 Pyruvate metabolism	0.049	Acetic acid Acetaldehyde Ethanol	6.2.1.1, 2.7.2.1, 1.1.1.1	0.16
map00720 Carbon fixation pathways in prokaryotes	0.01	Acetic acid Formic acid Succinic acid	6.2.1.1, 2.7.2.1 1.17.1.9 6.2.1.5	0.20
map00400 Phenylalanine, tyrosine, and tryptophan biosynthesis	0.00001	Phenylpyruvate	2.6.1.1, 2.6.1.57, 2.6.1.9, 4.2.1.51	0.38
map00450 Selenocompound metabolism	0.035	Methaneselenol	1.8.1.9	0.29
map00010 Glycolysis/gluconeogenesis	0.000004	Acetic acid Acetaldehyde Ethanol	6.2.1.1 1.1.1.1	0.36

KEGG pathway identifies which enriched pathway a given compound is found in. *p* value describes the significance (hypergeometric test) for enrichment of LUCA enzymes. bVOC lists the VOC being described, and enzymes indicate which enzyme in that pathway the VOC is associated with. Enzyme coverage describes the percent of unique enzymes in a pathway that were also found in our LUCA enzyme set

### Comparing the Modern vs Ancient Volatilome

Today, the largest contributor to global VOC fluxes, is isoprene, followed by terpenoids (Laothawornkitkul et al. 2009; Sindelarova et al. 2014; Guenther et al. 1995). Through our analysis, we see that both of these are absent from the predicted LUCA volatilome. Isoprene’s omission is possibly due to a limitation of available data. Isoprene is known to be produced across all domains of life, but with a high degree of differentiation between the primary producer, plants, and that of prokaryotic species (McGenity et al. 2018). Isoprene production in prokaryotes is not as well understood or characterized (particularly in archaea), and prokaryotic isoprene production enzymes are not found in KEGG. Previous literature has discussed the widespread evolutionary reach of isoprene production (Zhan et al. 2021). Due to incomplete data, we cannot say with certainty whether isoprene was produced by ancient life. Terpenoids are also absent from our results. The metabolism of secondary terpenoids is thought to have evolved relatively recently, following the GOE (Jia et al. 2022; Hoshino and Villanueva 2023). Our results complement this and demonstrate it is unlikely that volatile terpenoids were a component of LUCA’s volatilome.

The enrichment of LUCA VOCs among highly conserved metabolic functions, e.g., glycolysis, is expected given their ubiquitous nature and indicates a slimmed down LUCA volatilome. Among the VOCs that were predicted to be LUCA associated, many exist as intermediates in the production of other vital compounds. VOCs that are associated broadly across modern life, such as acetaldehyde, ethanol, acetic acid, and formic acid, were associated with LUCA. This indicates that LUCA may have not had the evolutionary freedom to invest in volatile secondary metabolite production. Or that, prior to land colonization, VOC production may have not conferred the same evolutionary benefit. Alternatively, early life may have produced secondary metabolites that were volatile, but given the bias of phylogenetic reconstruction toward highly conserved genes, these compounds are unable to be traced to LUCA in some of our source studies. Compounds which are not essential for primary metabolism (i.e., secondary) are less tightly bound by traditional selection pressures (Firn and Jones 2000). Even more complicating is an observed lack of enzyme specificity for some secondary metabolites (e.g., sesquiterpene synthase; Steele et al. 1998), making generalist enzymes simultaneously valuable and difficult sources for identifying which volatile metabolites may have been produced by early life. This highlights a limitation in attempting to explore volatile secondary metabolites on early Earth. Regardless, we find clear evidence that LUCA was metabolizing VOCs, albeit in a capacity far different from what is observed today. As plants and their sheer biomass constitute the dominant emission source of nearly all VOCs today (Laothawornkitkul et al. 2009), early Earth likely had a VOC emission capacity of predominantly small molecules (acetaldehyde, ethanol, etc.) produced by microorganisms which, barring extraordinary emission or biomass scenarios, would have generated net VOC emissions a fraction of modern levels. If this were the case, the widespread evolution of microbial life, the great oxygenation event, and the later advent of plant species may have all marked major transitions in the global volatilome.

### An Initial Framework for Early VOC Interactions

Here, we overview how our identified LUCA VOCs are cycled in modern systems and how this might inform our understanding of the development of global VOC cycling in the past, with the understanding that LUCA-associated metabolism may have existed in a range of environments. In the ocean, acetaldehyde is emitted predominantly by microalgae, likely as a result of cell lysis or as a passively diffusing metabolic intermediate (Pozzer et al. 2022), with resulting particle formation in the marine atmosphere. It is also ubiquitously consumed by heterotrophic ocean bacteria and used as a source of energy and carbon (Halsey and Giovannoni 2023), and modern net fluxes of acetaldehyde in aquatic systems can range from emissions to uptake. Some evidence indicates oxygenated VOCs formic acid and acetic acid may have emissions correlated with marine DOM in environments isolated from terrestrial inputs (Mungall et al. 2017), and ethanol has shown high variability in emissions from marine sources. At least one study has indicated net emission of ethanol from the ocean to the atmosphere, driven by biological production (Beale et al. 2010). Photosynthesis is thought to have evolved as early as 3.5 Gya (Blankenship 2010; Des Marais 2000), indicating a microbial community existed near the ocean surface well before the GOE. Phototrophic species near the ocean surface have been explored for their coupling with methanogens during the Archaean, allowing for a net methane flux from the ocean surface (Ozaki et al. 2018). Therefore, we can hypothesize that an early photosynthetic community near the ocean surface may have similarly facilitated VOC fluxes at the Archaean sea–atmosphere interface, resulting from VOC metabolism and photochemical degradation of metabolic products. The directionality of this flux may have been variable and dependent on the degree of abiotic VOC formation in the atmosphere relative to the level of biological activity. This, coupled with our established LUCA VOC metabolic potential, points to early oceans as clear sites for complex and consequential VOC cycling dynamics which may have begun early in Earth’s history. Terrestrial systems are well documented for a diverse suite of volatile metabolites. Among our most likely subset of LUCA VOCs, ethanol, acetaldehyde, acetic acid, and formic acid have measured emissions from modern soils (for example, Honeker et al. 2023). While LUCA is generally believed to have existed before the widespread microbial colonization of terrestrial surfaces, the presence of VOCs within LUCAs volatilome indicates that VOC cycling would have been a feature of terrestrial life from its onset.

Taken together, aquatic and terrestrial ecosystems couple to participate in dynamic microbially driven VOC fluxes to the atmosphere. Assuming that life originated in aquatic ecosystems, ocean inputs of VOCs to the atmosphere may have outweighed terrestrial inputs. For these ocean communities, VOCs may have been rapidly cycled within the marine system as low-molecular weight metabolites capable of passive absorption across cell membranes. As life expanded, increasing net VOC production could drive VOC emissions at the sea–atmosphere interface. Any VOCs released into the atmosphere would have an influence on atmospheric chemistry, for example impacting the lifespan of methane. We highlight that our list of LUCA-associated VOCs contain many highly water soluble compounds. Previous work has shown that in some anoxic exoplanet atmospheres, rainout and dissolution into oceans become the main sink for highly soluble compounds, preventing them from reaching high atmospheric concentrations in the absence of an extremely large emission source (Zhan et al. 2022; Huang et al. 2022). Given our identification of ancient VOC metabolites, it is worth considering that water solubility may have been a major feature deciding the fates of these compounds as opposed to atmospheric oxidation by OH radicals as seen today.

### A Note on Method Limitations

Studies on LUCA rely on diverse methods that can produce different results (Crapitto et al. 2022). Here, we rely on previous work published by Crapitto et al. (2022) to account for this using their complete list of LUCA-associated COGs across eight studies. However, an inherent limitation to this approach is the use of COG (a functional ortholog grouping tool) to describe phylogenetic results and interpret them through enzymatic classes. Converting from a functional category to enzymatic class is not a straightforward process. We relied on two separate methods to combat this. The first used Swissprot and may have limited the number of LUCA enzymes identified due to the smaller size of this database. Our second method allowed us to access the large KEGG database in an automated manner. However, we relied on simple linkages between KOs and COGs, as represented in the KEGG database. These linkages were not always one to one, and situations arose where one KO was associated with multiple COGs or vice versa. This may have biased toward KOs that were linked with many COGs and against those COGs with limited representation in the KEGG database. However, a benefit to this approach was in its automated nature. The ability to check for each KO in at least two phyla per prokaryotic species within the KEGG database allowed us to expand beyond the limitations of manual curation found in Swissprot and provide a more broader list of potential VOCs that would not be possible to produce manually. Using two separate COG to enzyme translation approaches each relying on a different source database (KEGG or Swissprot), we believe we provided a more robust analysis that still took advantage of the LUCA data summary provided by Crapitto et al. (2022) while being cautious in its interpretation of results. Still, simply confirming enzymes (in either Swissprot or KEGG) across domains may have overestimated an enzyme’s presence in LUCA as it did not account for lateral gene transfer (LGT). While some studies we relied on did take LGT into account (sometimes with controversial success–see Gogarten and Deamer 2016), it is possible that our conversion methods may have reintroduced some LGT products into the dataset. Phylogenetic tools do exist to mitigate this problem (Morel et al. 2020). However, our large dataset and reliance on KEGG for metabolite volatility estimations would have required the development of automated methods that do not currently exist and were beyond the scope of this study. We emphasize that this study was not an attempt at reconstructing LUCAs metabolome, but instead offering a novel interpretation of the diverse existing analyses on LUCA. While we attempted to mitigate potential drawbacks of our approach, we recognize that some inaccuracies may persist in our list of LUCA-associated enzymes and therefore VOCs. We also recognize that we relied heavily on enzyme-metabolite linkages found within the KEGG database. As described with isoprene, this may have introduced false negatives for metabolites with limited coverage in KEGG. For this reason, we encourage readers interested in the early metabolism of particular VOCs to keep this in mind when interpreting our results and incorporate additional metabolic databases for a more comprehensive perspective when needed.

## Conclusion

In this analysis, we identified VOCs associated with early life, from broad to conservative estimates. We show that, while early life did metabolize VOCs, this was likely limited to compounds involved in ancient, highly conserved metabolic pathways (e.g., amino acid biosynthesis and metabolism). The most dominant modern VOC sources (isoprene and terpenoids) were not associated with enzymes linked to LUCA across all reported studies, indicating a volatilome vastly different than today’s, with a noted depletion in volatile secondary metabolites. Other VOCs with high modern emissions, including acetaldehyde, acetic acid, formic acid, and ethanol were associated with LUCA across studies, showing that active biological VOC cycling on early Earth was possible, even in results from our more conservative volatile metabolite identification approach. VOC metabolism on early Earth may have begun even before terrestrial colonization and influenced atmospheric chemistry from its onset. Feedbacks between aquatic and terrestrial VOC cycling may have eventually led to a transition from a global volatilome dominated by oceanic metabolism at the sea–atmosphere interface to one dominated by terrestrial inputs as is seen today. The identification of these VOCs as associated with LUCA across studies demonstrates their function as early metabolites. Even if LUCA may have been constrained to a limited geographical scope, the presence of these VOCs as associated with LUCA indicates that as life diversified and expanded, these metabolites were maintained and actively cycled. High abiotic VOC production scenarios may have provided an additional source of organic carbon for energy and growth, while low abiotic production scenarios may indicate biology as a primary emission source. Whether as a source or sink, metabolism influenced VOC environmental chemistry with possible downstream impacts on global radiation balance, atmospheric oxidative capacity, and carbon cycling dynamics. Just as methanogenesis has been implicated as a form of metabolism with an important influence on early atmospheric chemistry, we argue that VOC metabolisms broadly should be similarly appreciated. Future work may use this information to explore particular VOC metabolites and constrain precisely how their metabolism by ancient life may have impacted phenomena, such as atmospheric oxidative capacity and nutrient cycling.

## Supplementary Information

Below is the link to the electronic supplementary material.

Supplementary file1 (XLS 757 KB)

Supplementary file2 (XLS 48 KB)

Supplementary file3 (XLS 877 KB)

Supplementary file4 (XLS 35 KB)

Supplementary file5 (XLS 21 KB)

Supplementary file6 (PDF 393 KB)

## Data Availability

The data supporting the findings of this study are available within the paper and its Supplementary Information files. Additional files may be provided upon request.
