# Supplementary material for: Volatile Organic Compound Metabolism on Early Earth
Source: J Mol Evol. 2024 Jul 17;92(5):605–17. doi: 10.1007/s00239-024-10184-x (PMC11458752; doi:10.1007/s00239-024-10184-x)
Supplement: Supplementary file 6 — Supplementary file6 (PDF 393 KB) [file 239_2024_10184_MOESM6_ESM.pdf]

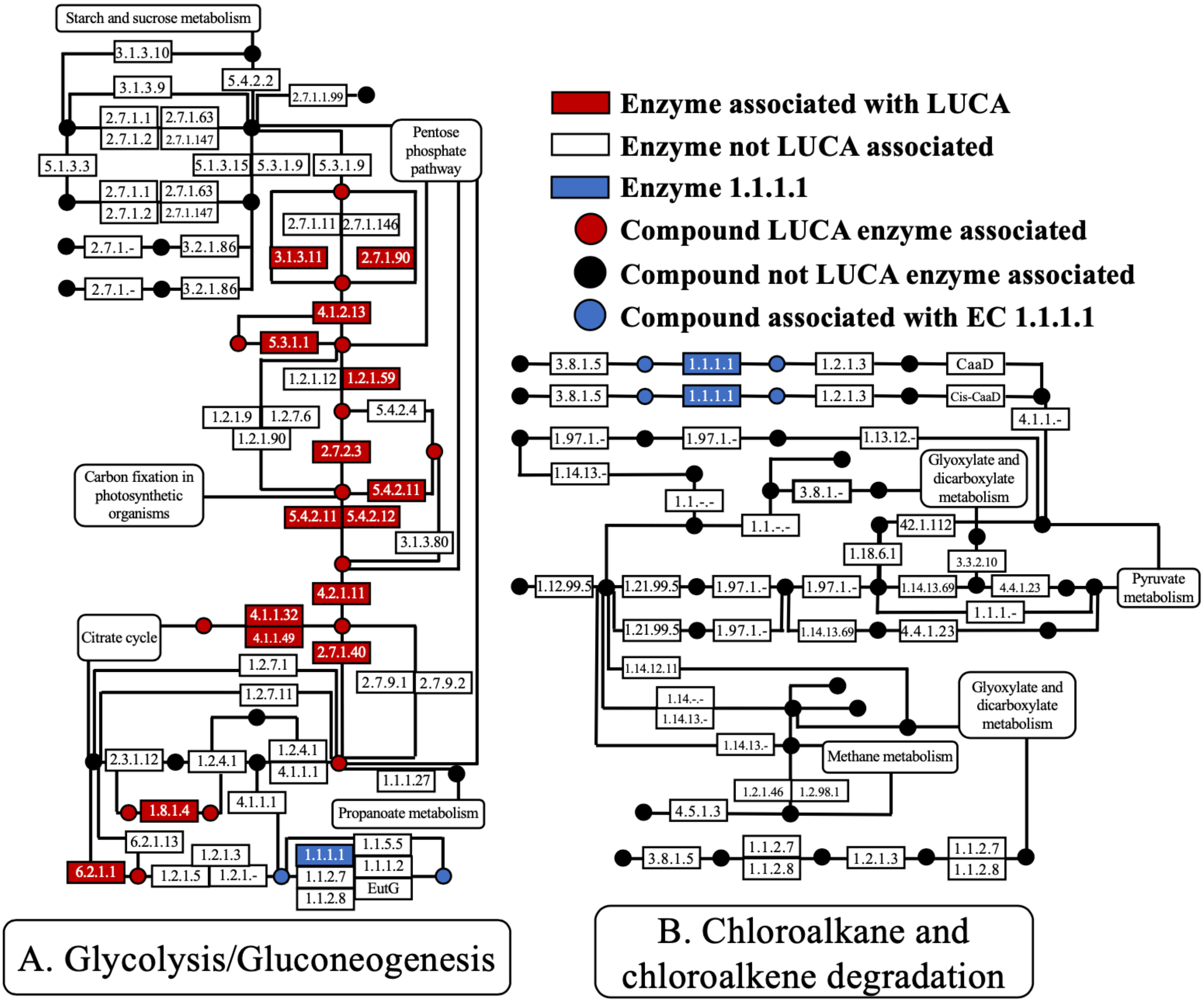

Supplemental Figure 1. Example of pathway enrichment for LUCA enzymes. The left pathway, Glycolysis/Gluconeogenesis, was significantly enriched for LUCA enzymes when running a hypergeometric test (p value = 0.000004). The pathway on the right was not. VOCs associated with EC 1.1.1.1 in the left pathway would be included as LUCA associated through our more conservative filtering process. VOCs associated with EC 1.1.1.1 would be associated with LUCA through our broad approach, but not through the more conservative filtering process.
